# Cost‐effectiveness of office‐based, magnetic resonance imaging‐guided transperineal versus transrectal prostate biopsy: An economic analysis of the PREVENT trial

**DOI:** 10.1002/cncr.70118

**Published:** 2025-10-28

**Authors:** Mitchell M. Huang, Conor B. Driscoll, Nicole Handa, B. Malik Wahba, Aaron A. Laviana, Hiten D. Patel, Ali Jalali, Jim C. Hu, Edward M. Schaeffer

**Affiliations:** ^1^ Department of Urology Feinberg School of Medicine Northwestern University Chicago Illinois USA; ^2^ Department of Urology Weill Cornell Medicine New York New York USA; ^3^ Department of Surgery and Perioperative Care The University of Texas at Austin Dell Medical School Austin Texas USA; ^4^ Surgery Service Jesse Brown VA Medical Center Chicago Illinois USA; ^5^ Department of Population Health Sciences Weill Cornell Medical College New York New York USA

**Keywords:** cost‐effectiveness analysis, infections, Markov model, prostate cancer, transperineal prostate biopsy

## Abstract

**Background:**

As antimicrobial resistance increases, safer alternative approaches to prostate biopsy are needed. PREVENT was a multi‐institutional, randomized controlled trial comparing transperineal (TP) biopsy without antibiotic prophylaxis versus transrectal (TR) biopsy with targeted prophylaxis. The authors conducted a secondary cost‐effectiveness analysis of PREVENT.

**Methods:**

The authors designed a Markov model with a simulated cohort of 1000 biopsied men. They assessed the short‐term cost‐effectiveness over a 2‐week period, comparing relative costs in US dollars and utility measured in quality‐adjusted life years (QALYs). The strategies they compared were office‐based, magnetic resonance imaging‐guided biopsy using two approaches: (1) TP without antibiotics; or (2) TR with targeted antibiotic prophylaxis. Analysis was from a health care payer perspective using a willingness‐to‐pay (WTP) threshold of $100,000/QALY. Probabilistic sensitivity analysis was performed with 5000 Monte Carlo simulations.

**Results:**

Compared to TR, TP was dominant, offering lower cost and higher utility per patient. This finding was robust to sensitivity analyses with TP having >89% probability of cost‐effectiveness regardless of WTP threshold. TP remained dominant when real‐world infection rates were used. TP biopsy needed to prevent >0.5% infections compared to TR to maintain cost‐effectiveness. Per 1000 patients, TP biopsy prevented 16 infections, and the additional cost to prevent a single infection was $3.18/patient.

**Conclusions:**

In this model, TP biopsy was more cost‐effective than TR from a health care payer perspective. In the setting of increasing concerns about the risk of infection from traditional TR biopsy, these findings suggest that office‐based TP biopsy is a more cost‐effective population‐level alternative.

## Abstract 2

A Markov model found that transperineal prostate biopsy was a more cost‐effective approach than transrectal. The per‐patient cost to prevent a single infection was $3.18.

## INTRODUCTION

Over 2 million biopsies are performed annually across the United States and Europe for prostate cancer detection.^1^, ^2^ Although prostate biopsy has traditionally been performed in a transrectal (TR) fashion with empiric antibiotic prophylaxis, there have long been concerns about the risk of serious infectious complications from this approach.^3^, ^4^, ^5^ Infection rates from TR biopsy of 5%–7% have previously been reported in the literature, with evidence that hospitalization and more serious infections have increased in recent years.^6^, ^7^, ^8^ This is likely driven by increasing fluoroquinolone resistance and has sparked increased interest in alternative strategies for prostate biopsy.^9^ One option is greater utilization of targeted prophylaxis for TR biopsy based on sensitivities from a rectal swab culture, which can lower rates of infection and sepsis.^10^, ^11^, ^12^

Another emerging alternative has been the transperineal (TP) approach to biopsy. Although TP biopsy was historically performed under general anesthesia, with concerns about acute urinary retention, recent technical advancements have led to the feasibility of an office‐based approach under local anesthesia.^13^ These developments resulted in increased discussion about the possibility of TP as an emerging standard‐of‐care approach to prostate biopsy.^14^ In fact, the current European Association of Urology (EAU) guidelines on urologic infections recommend that prostate biopsy be performed by a TP approach.^15^

The PREVENT trial was a multi‐institutional, randomized control trial comparing these competing approaches to preventing biopsy‐related infections.^16^ The study randomized patients with suspicious lesions on prostate magnetic resonance imaging (MRI) to receive office‐based, MRI‐guided biopsy by either a TP approach without antibiotic prophylaxis or TR with rectal swab‐targeted prophylaxis. In the final analysis after accrual targets were reached, there were no infections observed for TP versus an infection rate of 1.6% for TR.^17^ There were also fewer instances of urinary retention observed in the TP arm (0.3% TP vs. 1.1% TR).

Given the offsetting fixed procedural costs (rectal swab, antibiotics for TR vs. the cost of disposable equipment for TP) and the observed difference in complication rates and post‐procedural pain, it is unclear which approach is comparatively more cost‐effective. To this end, we conducted a secondary economic analysis of data from the PREVENT trial using a decision‐analytic model to evaluate the cost‐effectiveness of TP versus TR biopsy.

## MATERIALS AND METHODS

### Model design

**FIGURE 1:**
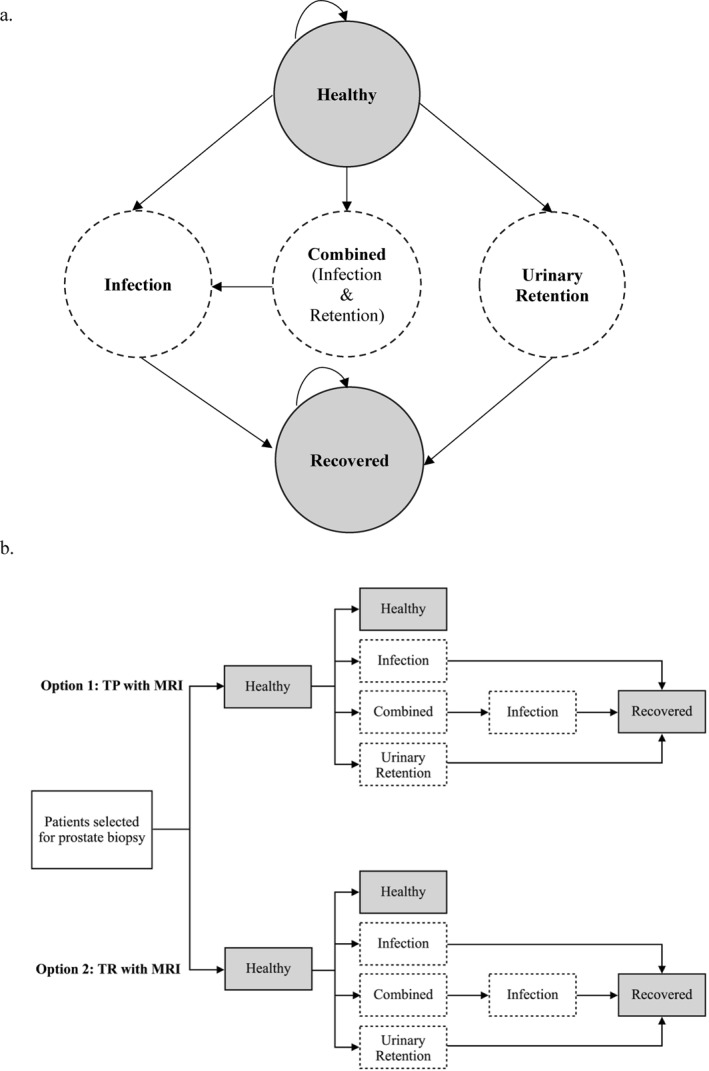
Markov model design (A) and decision tree (B) A five‐state Markov model was designed based on the most common complications observed in the PREVENT trial (A). A simulated cohort of men selected to undergo biopsy using either TP or TR strategies entered the model in the “Healthy” state that was assigned a utility of 1 (perfectly healthy) (B). Transitions to the lower utility “Infection” and “Urinary Retention” states occurred with each model cycle based on transition rates calculated using the complication probabilities observed in PREVENT (Table 1). Complication states were tunnel states (dashed circles/boxes), in which patients entered and then remained in the “Infection” state for seven cycles (7 days) and “Urinary Retention” state for three cycles before transitioning to “Recovered” where their utility returned to a baseline of 1. Patients remained in the “Combined” state for three cycles before transitioning to the “Infection” state, representing the time for retention to resolve. MRI indicates magnetic resonance imaging; TP, transperineal; TR, transrectal.

We simulated outcomes for a cohort of 1000 patients of PSA‐screening age (50–70 years old) selected to undergo prostate biopsy and compared the cost‐effectiveness of office‐based, MRI‐guided TP biopsy without antibiotic prophylaxis (“TP with MRI”) against TR biopsy with targeted antibiotic prophylaxis based on rectal swab (“TR with MRI”). We developed a decision‐analytic Markov model with five health states based on the two most common complications observed in PREVENT: “Healthy,” “Infection,” “Urinary Retention,” “Combined” (both infection and urinary retention), and “Recovered” (Figure 1). All patients entered the model in the Healthy state. The three complication states were tunnel states: patients entered the Infection state for seven cycles (i.e., 7 days) and Urinary Retention state for three cycles before transitioning to the Recovered state; patients entered the Combined state for three cycles before transitioning to the Infection state once the retention had resolved. Clinical parameters were derived from patient‐level data analysis reported in the PREVENT trial and are detailed in Table 1.^17^ Because no sepsis events were observed in PREVENT, similar to previous studies showing negligible sepsis rates for TP,^25^ we did not account for sepsis in our base model. Analysis was conducted from a health care payer perspective using a 2‐week time horizon and 1‐day cycles. Our model was created in Microsoft Excel v16.91 and Visual Basic for Applications programming was used to automate calculation.

**TABLE 1:** Model parameters and sources.

Parameter	Base model value	Source
Clinical parameters		
Probability of infection for TP (7 days)^a^	0%	PREVENT^17^
Probability of infection for TR (7 days)^a^	1.6%	PREVENT^17^
Probability of urinary retention for TP (7 days)^a^	0.3%	PREVENT^17^
Probability of urinary retention for TR (7 days)^a^	1.1%	PREVENT^17^
Duration of pain from procedure	1 hour	Assumption^b^
Cost parameters		
Prostate MRI	$466	CMS^18^
Prostate biopsy procedure fee	$2066	CMS^18^
Cost of rectal swab	$91	CMS^19^
Cost of prophylactic antibiotics	$5.51	FSS^20^
Cost of TP biopsy disposables	$218.92	Islam et al.^21^ ^c^
Cost of TR biopsy disposables	$57.03	Institution data
Cost to treat infection	$8399	FSS and CMS^d^
Cost to treat urinary retention	$1776	Wang et al.^22^
Cost to treat sepsis	$23,435	Gross et al.^23^
Utility parameters		
Baseline utility for healthy and recovered states	1.00	Assumption
Temporary utility for TP arm (pain)	0.83	PREVENT^16^ ^b^
Infection	0.74	Tufts CEAR^24^
Urinary retention	0.87	Tufts CEAR^24^
Sepsis	0.51	Tufts CEAR^24^

Abbreviations: CEAR, cost‐effectiveness analysis registry; CMS, Center for Medicare & Medicaid Services; FSS, federal supply schedule; MRI, magnetic resonance imaging; TP, transperineal; TR, transrectal.

^a^ Because our model had cycle lengths of 1 day, these probabilities (*p*) were converted into a daily rate (*r*) via the formula: *r* = –ln(1 – *p*)/*t*, where *t* was 7 days as no complications were observed after 1 week in PREVENT. The probability of daily recurrence was calculated as 1 – exp[–*r* × 1 day].

^b^ The pain disutility for the TP arm was calculated from PREVENT questionnaires that demonstrated a 0.6‐point difference in immediate post‐procedural pain (3.6 for TP vs. 3.0 for TR out of 10 points, a 1.2‐fold higher pain score); 0.83 was calculated by dividing a utility of 1 (perfect health) by 1.2. Given that we assumed that patients had 1 hour of greater pain from the procedure, the TP patients had a utility of 0.83 × 1/24 + 1 × 23/24 for cycle 1 of our model.

^c^ The cost of TP disposables included the condom, sensor cable, clip for the UroNav (Philips, Amsterdam, Netherlands) guide, and cost of PrecisionPoint (Perineologic, Cumberland, Maryland, USA) device. The PrecisionPoint device was found to cost $178 at our institutions that matches previously published prices (Islam et al.)^21^.

^d^ This was calculated by combining the cost of an emergency department visit, 2 days of inpatient admission, the cost of urine and blood cultures, 2 days of broad‐spectrum intravenous antibiotics, and then a 12‐day course of oral antibiotics.

### Utilities

Utility weights were assigned to each health state, with values ranging between 0 (representing death) and 1 (representing perfect health). Healthy and Recovered states were assigned a utility of 1. Utility weights for health states for urinary retention (mean, 0.87; SD, 0.03) and urinary tract infection (mean, 0.74; SD, 0.02) were obtained through the Tufts Cost‐Effectiveness Analysis Registry using previously published health utilities for these disease states.^24^ Because PREVENT reported that patients in the TP arm had a higher average pain score immediately post‐procedure (3.6 for TP vs 3.0 for TR out of 10 points, a 1.2‐fold higher reported pain), we assigned a temporarily lower utility for TP patients (0.83, derived by dividing a utility of 1, perfect health, by 1.2) for a portion of the first cycle of the model (1 hour, i.e., 1/24th of cycle 1) to account for the expected difference in procedure‐related pain.^16^

### Costs

Costs were assessed in US dollars ($) and assessed from a health care payer perspective and converted to 2024 dollars using the consumer price index. Costs for procedures, drugs, and laboratory studies were obtained through the Centers for Medicare and Medicaid Services and the Federal Supply Schedule.^18^, ^19^, ^20^ The cost of treating an infection was the sum total of facility costs (cost of emergency room [ER] visit, 2 days hospital stay), laboratory costs (blood and urine cultures), and treatment costs (broad spectrum IV antibiotics and fluids for 2 days and oral antibiotics for 12 days). The cost of treating urinary retention was calculated by a weighted average of previously published estimates for treating retention in the ER, urgent care, and urology clinic settings (each setting was weighted 1/3).^22^ The cost of prophylactic antibiotics for the TR arm was a weighted average calculated by multiplying the cost of each antibiotic by the proportion used in PREVENT.^16^, ^20^ Other costs included in the model are enumerated in Table 1.^21^, ^22^, ^23^ Given the short time horizon, we did not discount costs.

### Primary analysis

We assessed cost‐effectiveness of the two treatment options by calculating the incremental cost‐effectiveness ratio (ICER), which measures the per‐patient cost of an additional QALY gained. We employed a willingness‐to‐pay (WTP) threshold of $100,000/QALY for our primary analysis.^26^ Our secondary outcomes of interest were the total infections prevented, the total urinary retention episodes prevented, the cost to prevent a single infection, and the cost to prevent a single episode of urinary retention.

### Sensitivity analyses

We conducted one‐way sensitivity analyses in which model parameters were independently varied to identify the threshold where the ICER fell out of cost‐effectiveness range. For our probabilistic sensitivity analysis, we conducted 5000 Monte Carlo simulations in which all model parameters were simultaneously varied using beta distributions for clinical event probabilities and health utilities and gamma distributions for cost variables. When available, we used the variances reported in PREVENT to generate these distributions, otherwise the standard deviation was 20% of the baseline value. All WTP thresholds were tested from $0 until a treatment option had a 95% probability of being the more cost‐effective option.

We performed three additional, separate scenario‐based sensitivity analyses: 1) we included an additional arm, “TP Only,” to assess how omitting MRI impacted the cost‐effectiveness of office‐based TP biopsy. 2) We attempted to generalize our model using alternative clinical inputs from previous published literature; in these additional analyses, we included sepsis as a subset of the Infection health state, with a fraction of Infection patients experiencing lower utility and higher costs based on previously published literature.^23^, ^24^ We used rates published in large systematic reviews: a 0.31% infection rate and 0.09% sepsis rate for TP biopsy without antibiotic prophylaxis and a 0.72% infection rate and 0.48% sepsis rate for TR biopsy with targeted prophylaxis.^10^, ^27^ 3) We included an additional comparison arm “Empiric Antibiotics TR,” in which patients underwent TR biopsy and were given six doses of empiric oral ciprofloxacin without rectal swab. Sepsis was included as a subset of the Infection state; infection and sepsis rates for Empiric Antibiotic TR were set as 4.6% and 2.2%, respectively, based on a previous systematic review.^10^

## RESULTS

### Primary analysis

In our base model, TP with MRI was dominant versus TR with MRI, offering a lower per‐patient cost (–$83.62) and higher per‐patient utility (+0.0000705 QALY) over a 2‐week period (Table 2). Although TP with MRI had higher disposable costs than TR with MRI (net difference: +$161.90/patient), this was offset by the cost of rectal swab ($91/patient), antibiotics ($5.51/patient), and the higher cost of treating complications (net difference: +$134.67/patient for infection and +$14.34/patient for retention) in the TR with MRI arm. The TP with MRI cohort had 16 fewer infections and eight fewer cases of urinary retention than the TR with MRI cohort per 1000 patients. Compared to TR with MRI, the cost to prevent a single infection was $3.18/patient for TP with MRI; for every instance of urinary retention prevented, $8.58/patient was saved in the TP with MRI arm (Table 2).

**TABLE 2:** Base model (primary analysis).

	Transrectal	Transperineal
Costs (per patient)		
Fixed (e.g., MRI, procedure fee)	$2532	$2532
Disposables	$57	$218.89
Antibiotics	$5.51	—
Rectal swab	$91	—
Infection	$134.67	$0
Retention	$19.67	$5.33
Total	$2782.84	$2699.22
Utility (per patient)		
Total (QALY)	0.0410033	0.0410738
Incremental (vs. TR)		
∆ Cost	—	–$83.62
∆ Utility (QALY)	—	+0.0000705
ICER	—	Dominant
Clinical parameters		
Infections (per 1000 patients)	16.03	0
Urinary retention (per 1000 patients)	11.08	3.00
∆ Cost/infection prevented (per patient)	—	$3.18
∆ Cost/retention prevented (per patient)	—	–$8.58

Abbreviations: ICER, incremental cost‐effectiveness ratio; MRI, magnetic resonance imaging; QALY, quality‐adjusted life‐years; TR, transrectal.

### One‐way and probabilistic sensitivity analyses

**FIGURE 2:**
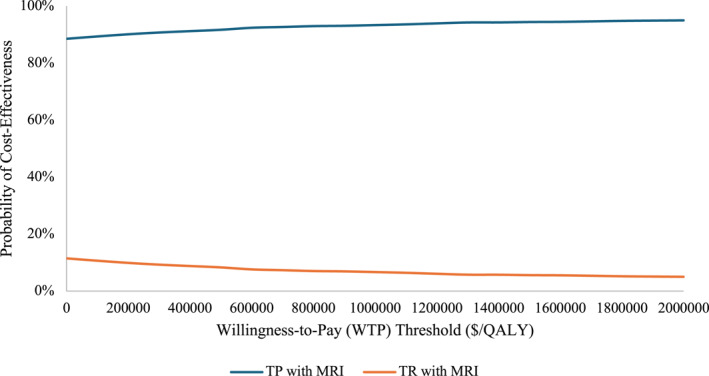
CEAC summary of the probabilistic sensitivity analysis, in which 5000 Monte Carlo simulations were performed with inputs varied simultaneously. Curves represent the probability of each strategy being the more cost‐effective option across different WTP thresholds from $0 to $2,000,000/QALY. CEAC indicates cost‐effectiveness acceptability curve; MRI, magnetic resonance imaging; QALY, quality‐adjusted life‐year; TP, transperineal; TR, transrectal; WTP, willingness‐to‐pay.

One‐way sensitivity analyses are summarized in Table 3. TP with MRI lost cost‐effectiveness under the following conditions: if TP biopsy prevented <0.6% fewer infections or resulted in >4.1% additional episodes of retention compared to TR. TP biopsy also became less cost‐effective if the difference in disposable costs was over $252.56, or if the cost of treating infection fell under $2744.06. The discrepancies in pain observed in PREVENT would need to be sustained at least 48 hours for TP to be less cost‐effective than TR biopsy. In our probabilistic sensitivity analysis, TP with MRI was more cost‐effective across all WTP thresholds examined (>89% probability of cost‐effectiveness), reaching 95% probability of cost‐effectiveness at a threshold of $1,700,000/QALY (Figure 2). Across all simulations, we found that TP with MRI outperformed TR with MRI as follows; median number of infections prevented: 15.3 per 1000 patients (interquartile range [IQR], 10.7–20.3), number of retention episodes prevented: 7.8 per 1000 patients (IQR, 3.6–12.7), cost per infection prevented: $3.09/patient (IQR, $1.33–$5.53), and savings per retention prevented: $5.82/patient (IQR, $0.18–$14.64).

**TABLE 3:** One‐way sensitivity analysis results.

Parameter	Threshold value
Clinical parameters	
Difference in probability of infection (TP vs. TR)	–0.5%
Difference in probability of retention (TP vs. TR)	+4.1%
Duration of pain from procedure	48 hours
Cost parameters	
Prostate MRI	None
Prostate biopsy procedure fee	None
Cost of rectal swab	None^a^
Cost of prophylactic antibiotics	None^a^
Difference in disposable costs (TP vs. TR)	+$252.57
Cost to treat infection	$2744.05
Cost to treat urinary retention	None^a^
Utility parameters	
Temporary utility for TP arm (pain)	0.21^b^
Infection	None
Urinary retention	None^a^

*Note:* The threshold value was the value at which TP biopsy was no longer cost‐effective using an ICER of $100,000/QALY. For comparisons of differences, all were from the frame of reference of TP biopsy compared against TR biopsy (e.g., for rate of infection, a –0.5% difference means a 0.5% lower rate of infection for TP biopsy compared to TR biopsy).

Abbreviations: ICER, incremental cost‐effectiveness ratio; MRI, magnetic resonance imaging; QALY, quality‐adjusted life‐years; TP, transperineal; TR, transrectal.

^a^ These calculated threshold values were impossible (i.e., negative cost or health utility over 1).

^b^ This translates to a 4.8‐fold higher pain score. Because TR biopsy had a mean score of 3.0 of 10 in PREVENT, this would be a mean 10 of 10 pain score for TP biopsy.

### Scenario‐based analysis 1: No MRI

When MRI was omitted from the TP arm, TP Only remained dominant over TR with MRI with a greater per‐patient savings of $549.62 and the same per‐patient difference in QALY (+0.0000705 QALY) versus the base model.

### Scenario‐based analysis 2: Systematic infection and sepsis rates

When using infection rates derived from systematic reviews of the literature and including sepsis, TP with MRI remained dominant over TR with MRI. Per 1000 patients, TP with MRI prevented 4.1 infections—including 3.9 cases of sepsis. The cost to prevent a single infection was $12.42/patient and the cost to prevent a single episode of sepsis was $13.07/patient.

### Scenario‐based analysis 3: TR with empiric antibiotic prophylaxis

Compared to Empiric Antibiotic TR as reference, both 1) TP with MRI and 2) TR with MRI were dominant. TR with MRI prevented 30 infections and 17 episodes of sepsis per 1000 patients at a cost of $3.05/patient to prevent an infection. TP with MRI prevented 46 infections and 22 episodes of sepsis per 1000 patients at a cost of $3.10/patient to prevent an infection (Table 4).

**TABLE 4:** Scenario analysis: transrectal biopsy with empiric antibiotic prophylaxis as reference arm.

	Reference: empiric antibiotics TR	Strategy 1: TR with MRI	Strategy 2: TP with MRI
Costs (per patient)			
Fixed (e.g. MRI, procedure fee)	$2532	$2532	$2532
Disposables	$57	$57	$218.89
Antibiotics	$4.68	$5.51	$0
Rectal swab	—	$91	—
Infection	$883.27	$206.98	$0
Retention	$19.83	$19.67	$5.33
Total	$3439.79	$2855.16	$2699.22
Utility (per patient)			
Total (QALYs)	0.0408479	0.041033	0.0410738
Incremental (vs. TR)			
∆ Cost	—	–$584.63	–$740.57
∆ Utility (QALYs)	—	+0.0001554	+0.0002259
ICER	—	Dominant	Dominant
Clinical parameters			
Infections (per 1000 patients)	46.09	16.03	0
Sepsis (per 1000 patients)	22.04	4.8	0
∆ Cost/infection prevented	—	$3.05	$3.10
∆ Cost/sepsis prevented	—	$5.32	$6.47

Abbreviations: ICER, incremental cost‐effectiveness ratio; MRI, magnetic resonance imaging; QALY, quality‐adjusted life‐years; TR, transrectal.

## DISCUSSION

In a cost‐effectiveness analysis based on the PREVENT trial, we found that for office‐based, MRI‐guided prostate biopsy, the TP approach was more cost‐effective than TR with targeted prophylaxis from a health care payer perspective. Our base model found that a TP with MRI strategy was dominant over TR with MRI, offering greater utility at lower cost. Our analysis found that the higher postoperative pain and upfront disposable costs for TP were outweighed by the higher incidence of complications and the additional cost of rectal swab and empiric antibiotics for TR biopsy. We found that for the TP approach, the additional cost to prevent a single infection was $3.18/patient when compared to TR. Our base model findings were also robust to alternative model inputs. TP biopsy maintained cost‐effectiveness for reductions in infection rate of at least 0.6% compared to TR. In our probabilistic sensitivity analysis, TP was more cost‐effective than TR biopsy in at least 89% of simulations for all WTP thresholds. These findings were not exclusive to the complication rates in PREVENT; TP biopsy remained dominant in our model when using previously published real‐world infection rates obtained from systematic reviews and when sepsis was included as a possible outcome.

To date, there have been several cost‐effectiveness studies comparing TP versus TR biopsy, both from the perspective of the United States health care system and the United Kingdom’s National Health System.^28^, ^29^, ^30^ Similar to the present study, all three reported that TP was more cost‐effective than TR biopsy. Two of these analyses additionally accounted for cancer detection in their analysis.^28^, ^30^ One used a longer time horizon of 10 years and included detection of metastatic disease.^29^ One analysis also noted that eliminating MRI further increased the relative cost‐effectiveness of TP biopsy.^30^ Our study similarly suggested that the cost‐effectiveness of TP is more pronounced if MRI is excluded, however such an approach could only be used if there were no detriment to the detection of clinically‐significant cancer. To our knowledge, ours is the first cost‐effectiveness analysis based on a randomized control trial specifically designed to compare the rates of infection and urinary retention between the two approaches. This is noteworthy given the quality of prospectively collected data compared to outcomes from retrospective studies in which post‐biopsy infection outcomes may be overestimated. Our study is also the first to include TR with targeted prophylaxis based on rectal swab as the comparison arm, an approach that reduces the rates of infection and results in improved antibiotic stewardship compared to empiric or augmented prophylactic strategies.^10^

Emerging antimicrobial resistance poses a significant risk not only to the health care system, but to society broadly.^31^ As fluoroquinolone and other antibiotic resistance increases, the risk of life‐threatening sepsis from TR prostate biopsy becomes a more salient consideration when making the decision to pursue a prostate biopsy.^3^ Concerns about infection have factored into previous decisions about guidelines surrounding PSA screening and could result in future limitations in prostate cancer screening if biopsy practices do not adapt.^4^ These considerations suggest a pressing need for improvements in biopsy strategy and help explain the increasing interest in TP biopsy.^14^ The findings of the present study suggest that on a population level, TP biopsy is the more cost‐effective alternative compared to TR with rectal swab‐targeted prophylaxis, primarily driven by differences in infection rate. Notably, our scenario‐based secondary analysis also demonstrates that when compared to a strategy of TR biopsy with empiric fluoroquinolone prophylaxis, TR with rectal swab‐targeted prophylaxis is dominant and prevents 30 infections per 1000 patients biopsied. This is an important observation, as empiric fluoroquinolone prophylaxis is still widely used across the United States and Europe.^32^, ^33^, ^34^ However, our findings do suggest that although targeted prophylaxis can make TR biopsy safer and more cost‐effective, a TP approach is the more economical strategy from a health care system perspective and the safer strategy for individual patients.

Our findings must be interpreted within the context of the study design. First, our model did not compare cancer detection between biopsy strategies as PREVENT did not show any significant difference in cancer detection.^16^ However, if reproducible differences in cancer detection are established, this may change the relative cost‐effectiveness of each approach. Second, our analysis was focused on the short‐term (2 weeks) and the outcomes of a single biopsy. This may fail to account for differences in longer‐term outcomes, including psychological burden of repeat biopsies, cancer‐related outcomes, and cumulative effects of repeat infections and antibiotic exposures. Conversely, because many patients in real‐world practice undergo multiple prostate biopsies—particularly those on active surveillance—the extent of TP’s relative cost‐effectiveness may be understated. Third, our model does make certain assumptions, most notably that the difference in pain between approaches resolved by 1 hour. However, PREVENT reported that all differences in pain resolved by 1 week and previous studies have demonstrated that discrepancies in pain are experienced primarily during the biopsy only.^16^, ^35^ Fourth, because our analysis was conducted from the perspective of the health care payer and was primarily an economic analysis, we did not account for the long‐term societal benefits of improved antibiotic stewardship and other noneconomic benefits.

In conclusion, our model found that office‐based, MRI‐guided TP biopsy was more cost‐effective than TR with targeted prophylaxis. TP was dominant in our base model and these findings were robust to alternative model inputs. TP was more cost‐effective in >89% of simulations across all WTP thresholds. The additional cost to prevent a single infection was slightly more than $3 per patient in the TP arm. Notably, our model does not account for the long‐term societal benefits of improved antibiotic stewardship that likely further favors TP biopsy.

## AUTHOR CONTRIBUTIONS

**Mitchell M. Huang**: Conceptualization; writing—original draft; writing—review and editing; formal analysis; software; data curation; validation; visualization; and methodology. **Conor B. Driscoll**: Conceptualization; writing—review and editing; supervision; and validation. **Nicole Handa**: Conceptualization; formal analysis; writing—review and editing; and validation. **B. Malik Wahba**: Conceptualization and writing—review and editing. **Aaron A. Laviana**: Conceptualization; writing—review and editing; supervision; and validation. **Hiten D. Patel**: Conceptualization; methodology; supervision; and validation. **Ali Jalali**: Supervision; project administration; writing—review and editing; formal analysis; and validation. **Jim C. Hu**: Conceptualization; resources; supervision; data curation; writing—review and editing; and investigation. **Edward M. Schaeffer**: Investigation; conceptualization; resources; supervision; project administration; and writing—review and editing; formal analysis.

## CONFLICT OF INTEREST STATEMENT

Nicole Handa reports fees for professional activities from Northwestern University Feinberg School of Medicine. Hiten D. Patel reports grant and/or contract funding from the Prostate Cancer Foundation. Edward M. Schaeffer reports consulting fees from Atria Academy of Science and Medicine, Early Medical, Lantheus Medical Imaging, Pfizer Canada Inc, and Pinnacle, Inc. The other authors declare no conflicts of interest.

## Data Availability

The data that support the findings of this study are available from the corresponding author on reasonable request.
